# Fixed Time-Point Analysis Reveals Repetitive Mild Traumatic Brain Injury Effects on Resting State Functional Magnetic Resonance Imaging Connectivity and Neuro-Spatial Protein Profiles 

**DOI:** 10.1089/neu.2022.0464

**Published:** 2023-09-29

**Authors:** Ravi Sakthivel, Marangelie Criado-Marrero, Daylin Barroso, Isadora M. Braga, Mackenzie Bolen, Uriel Rubinovich, Gabriela P. Hery, Matteo M. Grudny, John Koren, Stefan Prokop, Marcelo Febo, Jose Francisco Abisambra

**Affiliations:** ^1^ Center for Translational Research in Neurodegenerative Disease (CTRND), University of Florida, Gainesville, Florida, USA.; ^2^ Department of Neuroscience, University of Florida, Gainesville, Florida, USA.; ^3^ McKnight Brain Institute, University of Florida, Gainesville, Florida, USA.; ^4^ Department of Psychiatry, University of Florida, Gainesville, Florida, USA.; ^5^ Department of Pathology, University of Florida, Gainesville, Florida, USA.; ^6^ Fixel Institute for Neurological Diseases, University of Florida, Gainesville, Florida, USA.; ^7^ Brain Injury Rehabilitation and Neuroresilience (BRAIN) Center, University of Florida, Gainesville, Florida, USA.

**Keywords:** CHIMERA, diffusion tensor imaging, microglia, optic tract, repetitive mild TBI, resting state fMRI, thalamus

## Abstract

Repetitive mild traumatic brain injuries (rmTBIs) are serious trauma events responsible for the development of numerous neurodegenerative disorders. A major challenge in developing diagnostics and treatments for the consequences of rmTBI is the fundamental knowledge gaps of the molecular mechanisms responsible for neurodegeneration. It is both critical and urgent to understand the neuropathological and functional consequences of rmTBI to develop effective therapeutic strategies. Using the Closed-Head Impact Model of Engineered Rotational Acceleration, or CHIMERA, we measured neural changes following injury, including brain volume, diffusion tensor imaging, and resting-state functional magnetic resonance imaging coupled with graph theory and functional connectivity analyses. We determined the effect of rmTBI on markers of gliosis and used NanoString-GeoMx to add a digital-spatial protein profiling analysis of neurodegenerative disease-associated proteins in gray and white matter regions. Our analyses revealed aberrant connectivity changes in the thalamus, independent of microstructural damage or neuroinflammation. We also identified distinct changes in the levels of proteins linked to various neurodegenerative processes including total and phospho-tau species and cell proliferation markers. Together, our data show that rmTBI significantly alters brain functional connectivity and causes distinct protein changes in morphologically intact brain areas.

## Introduction

Repetitive mild traumatic brain injuries (rmTBIs) are the most frequently diagnosed form of head injury in the United States.^1–4^ These repetitive injuries can occur following multiple and subsequent impacting blows to the head—impacts such as those common to persons engaging in contact sports and by active military personnel in hazardous environments. Moreover, evidence suggests that rmTBI significantly increase the risk of developing neurodegenerative disorders, including chronic traumatic encephalopathy (CTE), Alzheimer's disease (AD), and other AD-related disorders (ADRD), more than some other forms of traumatic brain injury (such as those received following a single impact).^5–9^

The mechanism or mechanisms linking the physical disruption and damage of rmTBI to a subsequent pathogenesis of neurodegenerative disease remain largely unknown. However, advances in our ability to model and monitor the anatomical and molecular changes will help bridge our knowledge gaps. For example, diffusion tensor imaging (DTI) in magnetic resonance imaging (MRI) is capable of detecting microstructural changes, such as those caused by shearing forces and are responsible for diffuse axonal injury (DAI), a typical sign of rmTBI.^10^ Post-injury gliosis can be monitored by the increased expression of the microglial marker, ionized calcium-binding adapter molecule 1 (Iba1), and the astrocytic marker, glial fibrillary acidic protein (GFAP). Although these network and molecular characterizations can reveal considerable mechanistic information, none of these data are established as early options to assess and diagnose rmTBI. A goal of these studies is to lend weight to these analyses as early diagnostic tools while gleaning data relevant to disease pathology downstream of rmTBI. Additionally, because mild TBI symptoms can manifest days or months post-injury (often being unrecognized or improperly treated) causing long-term health issues, all analyses in this study were performed seven days after rmTBI to model a reasonable assessment or diagnostic window for persons that experienced rmTBI.

In this study, we used functional and static brain imaging, immunohistochemistry, and digital spatial protein profiling to characterize the physical and physiological changes occurring after rmTBI. In these analyses, we placed special emphasis on white matter regions, which are particularly susceptible to rmTBI.^11^ We identified hyper-specific regional abnormalities resulting from rmTBI by means of MRI-derived network analyses, traditional immunohistochemistry, and spatial proteomic analysis. These data support ongoing efforts to enhance the development of both imaging and protein biomarker techniques for diagnosing, monitoring, and pathologically understanding the progression and consequences of a TBI.

## Methods

### Animals

Male and female C57BL/6J (WT) mice were purchased from the Jackson Laboratory (Bar Harbor, ME). Two to five mice of same-sex groups were housed in standard room conditions with 12 h light and dark cycle and allowed to access free food and water *ad libitum.* The total number of mice used after sham or injured procedures was: 20 mice (15 male; five female) in the rmTBI group and 15 mice (13 male; two female) in the sham group. All animal procedures were approved by the University of Florida's Institutional Animal Care and Use Committee.

### Repetitive mild traumatic brain injury (rmTBI) by Closed-Head Impact Model of Engineered Rotational Acceleration

The rmTBI procedure was followed as suggested by Namjoshi and colleagues 2014; 2.5-3 months old male and female WT mice were subjected to two mild closed-head injuries (0.6 J) at 24 h intervals using Closed-Head Impact Model of Engineered Rotational Acceleration (CHIMERA) impactor. Number and temporal spacing of injuries mimic our previous findings using a closed-head injury model of TBI.^12^ Mice were anesthetized with isoflurane (induction 3-4% and maintenance 1-2.5%). Meloxicam (10 mg/kg body weight) was administered subcutaneously for 2 days prior to impact procedure to mitigate pain in the test animals. Animals were positioned supine in the holding bed of the impactor. After impact, animals were transferred immediately to the recovery chamber until fully ambulatory. Sham mice were exposed to all these procedures, except for the impact. At 7 days post-injury (dpi), transcardial perfusions were performed with sterile ice-cold 0.9 % saline and the brain tissues were harvested. One hemi-brain was micro-dissected and stored for further analysis. The remaining hemi-brain was fixed in 10% neutral buffered formalin for further histological analysis.

### T2 and resting-state functional magnetic resonance imaging data acquisition

Anatomical T2-weighted, diffusion weighted and resting-state functional magnetic resonance imaging (rsfMRI) scans were collected between 5-7 dpi for rsfMRI and DTI analyses. Sham and rmTBI mice were anesthetized with a mixture of isoflurane (induction 4%, maintenance: 1%) and medical grade air (70% N_2_ and 30% O_2_) and positioned prone on a custom-built holder with a radiofrequency (RF) coil was placed atop the head. Images were acquired using an 11.1T MRI scanner (Magnex Scientific Ltd, Oxford, UK) with high power gradient sets (RRI BFG-240/120-S7; maximum gradient strength of 1000 mT/m at 325 Amps and a 200 msec rise time; RRI, Billerica, MA). A custom-made 2.0 cm × 2.5 cm quadrature RF surface transmit/receive coil tuned to 470.7 MHz (1 H resonance) was used for B1 excitation and signal detection (RF engineering lab; Advanced Magnetic Resonance Imaging and Spectroscopy Facility, Gainesville, FL).

For rsfMRI study, a T2- weighted Turbo Rapid Acquisition with Refocused Echoes (TurboRARE) sequence was acquired with the following parameters: effective echo time (TE) = 41.42 msec, repetition time (TR) = 4 sec, RARE factor = 16, number of averages = 12, the field of view of 15 mm × 15 mm and 0.9 mm thick slice, and a data matrix of 256 × 256 with 14 interleaved ascending coronal slices. Resting-state images were collected using a single-shot spin-echo echo planar imaging (SE-EPI) sequence with the following parameters: TE = 15 msec, TR = 2 sec, 600 repetitions, and a data matrix of 64 × 48 with 14 interleaved ascending coronal slices in the same space as the T2 anatomical. Two additional single repetition SE-EPI scans were collected, one with phase encode gradient lobes (“PE blips”) collected along the positive gradient direction and the other collected with PE blips reversed to the negative PE direction (Bruker Paravision binary method for distortion correction is kindly provided by Dr. Matthew Budde, Medical College of Wisconsin).

For DTI images, 4-shot, 2-shell spin echo planar diffusion imaging sequence in Bruker Paravision, with TR = 4 sec, TE = 19 msec, number of averages = 4, gradient duration δ = 3 msec, diffusion time Δ = 8 msec, 54 images with three different diffusion weightings, two b = 0, 6 directions with b = 600 sec/mm^2^, and 46 directions with b = 2000 sec/mm^2^. Image saturation bands were placed on either side and below the brain to suppress non-brain signal during image acquisition. A navigator signal was used by the Bruker reconstruction software to improve signal stability in the 4-shot EPI. Diffusion images had the same field of view (FOV) and slice thickness as the anatomical scan but with a lower resolution data matrix size of 128 × 96 and 17 slices (resolution: 0.117 mm × 0.117 mm × 0.7 mm) in the same space as anatomical scans.

### rsfMRI and DTI image pre-processing and analysis

Functional connectivity of rmTBI mice brain was analyzed by graph theoretical analysis.^13^ SE-EPI distortions were corrected using TOPUP in FSL.^14^ The pair of opposite phase-encode blip scans were used to estimate the susceptibility-induced off-resonance field, which is used to unwarp subsequent EPI volumes.^14^ Image pre-processing and analysis was carried via in-house UNIX terminal using Analysis of Functional NeuroImages (AFNI),^15^ FMRIB Software Library (FSL)^16-18^ and Advanced Normalization Tools (ANTs).^19,20^ Anatomical and distortion-corrected functional scan masks outlining mouse brain boundaries were generated in MATLAB using Three-Dimensional Pulsed Coupled Neural Networks (PCNN3D).^21^ 3dDespike in AFNI was used to remove time series spikes and 3dvolreg for image volume alignment. Preprocessed scans were cropped and a high-pass temporal filter (< 0.009Hz) was used (3dTproject) to remove slow variations (temporal drift) in the fMRI signal. Independent component analysis decomposition was then applied using Multivariate Exploratory Optimized Decomposition into Independent Components (FSL MELODIC version 3.0) to preprocessed scans to assess noise components in each subjects' native space prior to spatial smoothing and registration. Noise-related signal along brain edges, in ventricular voxels, and large vessel regions were suppressed using a soft (“non-aggressive”) regression approach using fsl_regfilt.^16^ A low-pass filter (> 0.12 Hz) and spatial smoothing (0.4 mm full width at half maximum [FWHM]) were then applied to the fMRI scans.

Preprocessed anatomical and fMRI scans were aligned to a parcellated mouse common coordinate framework (version 3, or CCFv3) template.^22^ Bilateral region of interest (ROI)-based nodes (64 total) were created with the guidance of the annotated CCFv3 parcellation and using tools in ITKSNAP and FSL. In ITKSNAP, the template with overlaid parcellation was used to find the left hemisphere voxel coordinates for each of the nodes included in this study, which were distributed as evenly as possibly without overlap across the mouse brain template. The node coordinates were positioned in subregions of all areas for example the optic tract and thalamus. We created 0.6 mm diameter spherical nodes (resolution: 0.05 mm^3^) centered on the voxel coordinates. The right hemispheric representations of the same nodes were then created to complete left and right representations for each node. For subject-to-atlas registration, fMRI scans are up-sampled from a native space 0.234 × 0.3125 × 0.9 mm resolution (spatially smoothed at 0.4 mm FWHM) to a down-sampled template resolution of 0.1 mm^3^.

Anatomical scans were cropped and N4 bias field correction^23^ applied to T2 images to correct intensity variations due to RF field inhomogeneities.^24^ The extracted brain maps were linearly registered to the mouse template using FSL linear registration tool (FLIRT).^16^ The linear registration output was then nonlinearly warped to the template space using ANTs (antsIntroduction.sh script). Anatomical-to-atlas linear and nonlinear transformation matrices were applied to fMRI scans at a later stage. Brain extraction using a mask was first applied to fMRI scans and the cropped scans were then aligned to their respective higher resolution anatomical scans. Timeseries functional images were split into 600 individual volumes and the first in the series was linearly aligned to the anatomical scan using FLIRT (same parameters as above, except 6 degrees of freedom was used in this step). ANTs (antsRegistrationSyNQuick.sh script) was used to warp the lower resolution functional images to their structural (using a single stage step deformable b-spline syn with a 26-step b-spline distance). Linear and nonlinear warping matrices for fMRI-to-anatomical alignment were applied to individual scans in the time series, then the merged 4-D functional timeseries were moved to the atlas space using the prior anatomical-to-template transformation matrices.

A total of 64 ROI masks, divided into 32 left and 32 right ROIs, were included in our analyses. Center voxel coordinates were used for 3D network visualizations in BrainNet viewer in MATLAB.^25^ Signal timeseries were extracted resulting in 64 individual ROI text files per subject with L2-normalized resting state signals as a vector of 600 data points. The timeseries files were used in cross-correlations and in calculations of Pearson r coefficients for every pairwise combinations of ROIs (1dCorrelate in AFNI). The resulting number of pairwise correlations was 1952 per subject (after removing 64 self-correlations). Correlation coefficients were imported to MATLAB and Fisher's transform applied to ensure a normal distribution of z values prior to analyses.

Diffusion MRI scans were processed as previously described using tools FMRIB software library FSL.^26,27^ Tensor element reconstruction and estimates was performed using weighted least squares regression on DTIFIT in FSL.^28^ Regions of interest were manually selected in ITK-SNAP to estimate their mean intensity and volumes. These included thalamus, optic tract, fimbria, corpus callosum, hippocampus, and amygdala.

### Functional network analysis

Functional network analysis was completed as previously reported.^29^ Briefly, the Brain Connectivity Toolbox^30^ and MATLAB were used to determine weighted matrices. Edge densities thresholds were set in a range from 2 to 40% to calculate the following global network metrics: Clustering Coefficient (tendency of nodes to cluster and connect within the network), Characteristic Path Length (average of shortest path length between all pairs of nodes in the network), Transitivity (probability of neighboring nodes to be interconnected within the network), Global Efficiency (efficiency to communicate across distant brain regions), Louvain Modularity (density of connections within a neural cluster), and Small World Index (high local clustering with shortest path length). Unless otherwise indicated in the figures, a 10% threshold was used for all the node-specific measures.^13,29^

### Immunohistochemistry

Formalin-fixed, Paraffin-embedded coronal brain sections (5 μm) were used for the immunohistochemistry (IHC) study. Both sham and rmTBI slides were processed simultaneously. The slides were deparaffinized in xylene twice for 5 min each and rehydrated sequentially in the gradient of ethanol followed by water for 3 min each. The antigen retrieval was performed by immersing slides in 10 mM citrate with 0.5% Tween 20 (pH 6.0) and 30 min incubation in the steamer. The endogenous peroxidases were removed by incubating the slides with the mixture of 0.3% H_2_O_2_/phosphate-buffered saline (PBS) and 10% Triton X-100 (100 μL for 200 mL of 0.3% H_2_O_2_ solution) for 20 min. Blocking was performed with 10% normal goat serum/phosphate-buffered saline with Tween (PBS-T; 0.05% Tween) for 30 min at room temperature (RT). After blocking, the brain sections were incubated overnight with the following antibodies: Iba1 (1:1000, PA5-27436, Invitrogen) and GFAP (GA5; 1:1000, 3670S, Cell Signaling) at 4°C for microglia and astrocytes, respectively. After incubation, the slides were washed with PBS-T and incubated for 30 min with corresponding biotinylated secondary antibody (Goat Anti-Rabbit IgG Antibody [H+L], Biotinylated-[BA-1000]; Goat Anti-Mouse IgG Antibody, Biotinylated, R.T.U. BP-9200; Vector Laboratories).

Further, the sections were incubated with avidin-biotin complex (ABC) reagent for 20 min (VECTASTAIN Elite ABC-HRP Kit, Peroxidase Standard), PK-6100) following washes with PBS-T and developing with 3, 3′-diaminobenzidine (DAB) kit (KPL DAB Reagent Set, SeraCare cat no. #5510-0031). The slides were then washed twice in PBS, 5 min each, and counter-stained with hematoxylin for 1-2 min. The slides were washed briefly in water and dehydrated with the gradient of alcohol (70% ethanol, 90% ethanol, and 100% ethanol) for 3 min each and incubated in xylene twice for 5 min each. The sections were mounted with Cytoseal Mountant 60 (Epredia 83104) and air dried. The slides were scanned using an Aperio image scanner (20 × objective lens, Scan Scope™ XT, Aperio Technologies, Inc. Vista, CA) and quantified using Image Scope software (v12.4.3.5008) positive pixel count program. Three sections per mouse at 55 μm apart were averaged to calculate the expression of Iba1 and GFAP.

### Spatial proteomics analysis using the NanoString GeoMx Digital Spatial Protein profiling (DSP) platform

Formalin-fixed paraffin-embedded brain tissues were sectioned on the coronal plane at 5 μm thickness. DSP staining and assay procedures were conducted according to the manufacturer-directed protocol. Nuclei were fluorescently labeled with SYTO 13 (GeoMx Nuclear Stain Morphology kit, Item no. 121300303, Nanostring, Seattle, WA), and microglia cells were labeled with an Iba1 (GeoMx Alzheimer's Morphology Kit, Item no. 121300306, NanoString, Seattle, WA) antibody. The regions of interest (ROIs) were selected based on visual identification of anatomic regions using SYTO 13, aided by Iba1 expression. The following ROIs were selected: primary somatic sensory area, dentate gyrus polymorph layer, thalamus, and the optic tract. The following protein cores and modules were used for multiplexed protein quantification: Neural Cell Profiling (Item no. 121300120), Parkinson's disease pathology (Item no. 121300122), Alzheimer's disease (AD) pathology (Item no. 121300121), AD pathology extended (Item no. 121300123), Glial cell subtyping (Item no. 121300125), and autophagy (Item no. 121300124) markers. Samples were processed on the NanoString GeoMx system and the nCounter max system following manufacturer's instructions. Data were analyzed using GeoMx DSP analysis suit Version 2.4.0.421 as suggested by manufacturer instructions. Briefly, the Field of view registered-quality control (FOV-QC) was adjusted to 45% to check the low count score. All the QC passed segments were normalized to housekeepers such as glyceraldehyde 3-phosphate dehydrogenase and histone H3. Background corrections were performed with the negative background targets. Background corrected files were changed to tab-delimited files, and custom scripts with slight modifications were used to generate volcano plots.

### Statistical analysis

GraphPad Prism 9 software was used to perform all the statistical analysis. Mann-Whitney test followed up with FDR-Benjamini Hochberg correction (q = 5%) to determine significant differences in nodal properties between control and injured mice. Grubbs' test was performed (Alpha = 0.05). Outliers, data beyond the outlier boundary with mean ± 1.5 standard deviations, were removed from global, regional, and efficiency analyses data sets. Repeated measures two-way analysis of variance was performed to measure the global differences and differences between density thresholds in rsfMRI data. An unpaired t-test was performed within the GeoMx DSP analysis suit to analyze the NanoString GeoMx Spatial Profiling data. Uncorrected P-values and effect size are reported using methods previously described for functional connectivity network visualizations.^29^ All the results were represented as mean ± standard deviation; *p* < 0.05 is considered statistically significant.

## Results

**FIG. 1.**
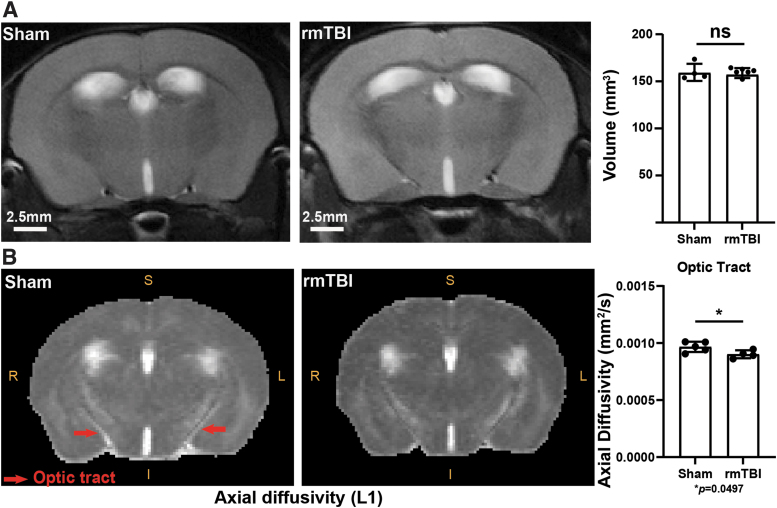
Volume and axonal integrity assessment following repetitive mild traumatic brain injury (rmTBI). **(A)** Representative T2-weighted brain images of sham and rmTBI mice used for brain volume analysis and quantification of volume from captured images **(A)**. Representative images of optic tract used in diffusion tensor imaging (DTI) microstructure pattern analysis of axial diffusivity (L1) and results with statistical analysis **(B)**. Units for diffusivity: mm^2^/sec; *p = 0.0497, unpaired t-test, mean ± standard deviation.

Animal injuries and outcomes were performed on a fixed schedule. We found no macrostructural or volumetric changes in the injured mice compared with controls (Fig. 1A); a finding consistent with previous reports on the CHIMERA model.^3,31^ In the absence of macro-scale changes to volume, we examined changes or damage on the microstructural level using DTI (diffusion of water molecules) in several key regions: thalamus, optic tract, corpus callosum, peri-aqueductal gray matter, hypothalamus, third ventricle, anterior cingulate area, infralimbic cortex, prelimbic cortex, anterior commissure, lateral ventricle, and cerebral peduncles (Supplementary Fig. S1). We observed that the axial diffusivity was significantly decreased in the optic tract of the rmTBI group (Fig. 1B; L1, *p = 0.0497), indicating a microstructural disruption of white matter in this region. We did not detect DTI changes in other monitored regions. No changes in fractional anisotropy, mean diffusivity, and radial diffusivity were observed in the rmTBI group (negative data not shown). Structural disruption of the optic tract following CHIMERA is in line with previous reports.^32^

**FIG. 2.**
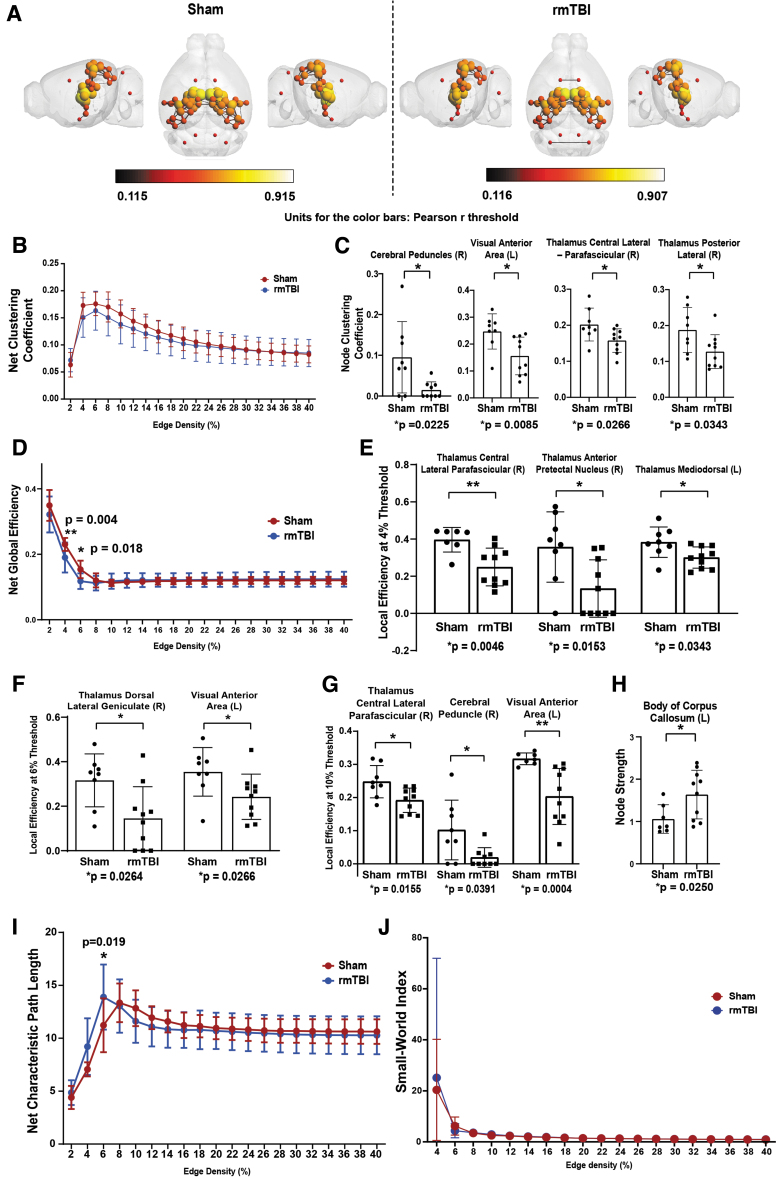
Clustering coefficient, global, and local efficiency analyses reveal network integration and efficiency changes following repetitive mild traumatic brain injury (rmTBI). R = right hemisphere; L = left hemisphere. 3D functional connectome maps position of nodes and edges in the region of interest (ROI; total ROI = 64); units for the color bars: Pearson r threshold **(A)**. Global **(B)** and local **(C)** clustering coefficient (the measure of regionalized connectivity of an individual node). Global efficiency **(D)** and local efficiency at 4, 6, and 10% thresholds (**E-G,** respectively). Node strength (Strength of total nodal connections per node) at 10% threshold **(H)**. Global path length, the measure of average distance between all potential pairs of nodes in a network **(I)**, and Small-World Index **(J)** analyses. The global (Net) analyses were performed using two-way analysis of variance, mean ± standard error at edge densities ranges 2 to 40%. The local nodal properties between sham and rmTBI was analyzed by Mann-Whitney test; mean ± standard deviation.

Due to the absence of major structural disruptions after CHIMERA, we surveyed functional changes in areas devoid of significant structural damage. To this end, we used several rsfMRI analyses associated with nodal connectivity and function. These analyses were applied to 64 nodes (Supplementary Table S1) that are closely associated with white matter tracts (Fig. 2A). No changes were observed in the net clustering coefficient (Fig. 2B). However, local regions presented differences between the two experimental groups that were statistically significant, including the cerebellar peduncle (*p* = 0.0225, 0.5470), the visual anterior region (*p* = 0.0085, Q = 0.5470) and two thalamic regions, the central lateral parafascicular and the posterior lateral region (*p* = 0.0266, 0.5485 and *p* = 0.0343, 0.5485; Fig. 2C).

Additionally, we assessed the efficiency of all nodes: individually and integrated as a group. We noted significant decreases at the 4% and 6% threshold in the rmTBI brains (*p* = 0.004 and *p* = 0.018, respectively; Fig. 2D). Specific regions presenting statistical differences at the 4%, 6%, and 10% thresholds were primarily noted in thalamic and visual regions; including the thalamic central lateral parafascicular (4%: *p* = 0.0046; 10%: *p* = 0.0155), thalamic anterior pretectal nucleus (4%: *p* = 0.0153), thalamic mediodorsal (4%: *p* = 0.0343), thalamic dorsal lateral geniculate (6%: *p* = 0.0264), the visual anterior area (6%: *p* = 0.0266; 10%: *p* = 0.0004), and the cerebral peduncle (10%: *p* = 0.0391; Fig. 2E-G). One node, the body of the corpus collosum, was identified as having a statistically significant elevation in node strength in the rmTBI animals (Fig. 2H). The net characteristic pathlength, revealing functional network integration, was significantly increased (*p* = 0.019) in the rmTBI group at exactly 6% edge density (Fig. 2I). The small-world index, an assessment of high clustering nodes with low minimal pathlengths, showed no differences between the two groups (Fig. 2J).

**FIG. 3.**
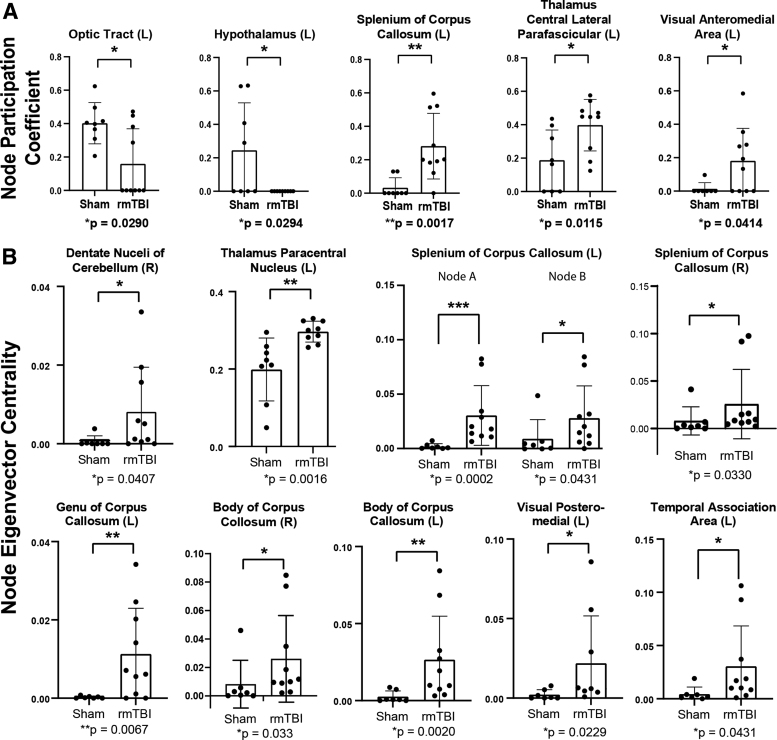
rmTBI disrupted connectivity between modules and increased eigenvector centrality. Participation coefficient measures the interaction of nodes in a module with those of a different module **(A)**. Eigenvector centrality assesses highly influential nodes within a network **(B)**. All analyses were Mann-Whitney tests; displayed as mean ± standard deviation.

To further examine inter-region connectivity, we measured node participation coefficients (the interaction of the nodes within each other and with other modules) and eigenvector centrality (identification of highly influential nodes within a network). Participation coefficient deficits in rmTBI mice, relative to sham mice (*p* < 0.05, Cohen's │d│ ≥ 0.8), were identified in the optic tract (*p* = 0.029) and the hypothalamus (*p* = 0.0294). Conversely, the participation coefficient of several nodes was increased in rmTBI mice, including the splenium of the corpus callosum (*p* = 0.0017), the thalamic central lateral parafascicular (*p* = 0.0115), and the visual anteromedial area (*p* = 0.0414; Fig. 3A).

Calculations into the eigenvector centrality of the nodes' impact across a network revealed elevated nodal influence in several regions from rmTBI mice (relative to sham mice) including the dentate nuclei of the cerebellum (*p* = 0.0407), thalamic central lateral parafascicular (*p* = 0.0016), splenium of the corpus callosum (left [L]-A *p* = 0.0002; L-B *p* = 0.0431; right (R) *p* = 0.0330), the genu of the corpus collosum (*p* = 0.0067), the body of the corpus collosum (R *p* = 0.033; L *p* = 0.002), the visual posteriomedial area (*p* = 0.0229) and the temporal association area (*p* = 0.0431; Fig. 3B). Other network features, such as global network strength, Louvain modularity, assortativity, transivity, gamma, and lambda values, showed modest or undetectable differences between the sham and rmTBI groups that were not significantly different (Supplementary Fig. S2). 

**FIG. 4.**
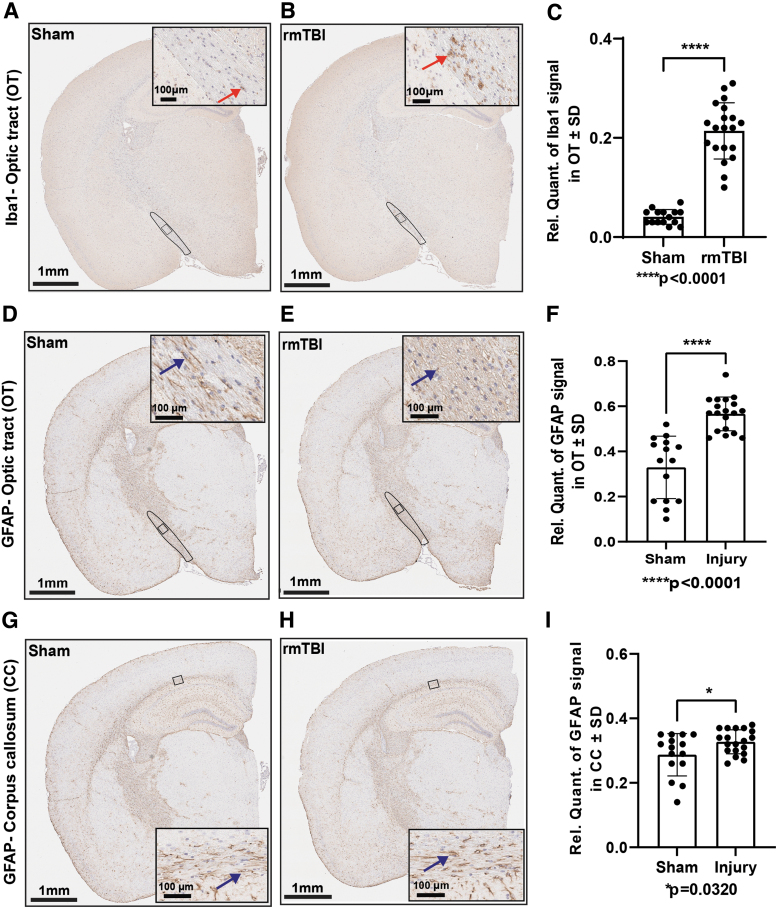
Repetitive mild traumatic brain injury (rmTBI) induced gliosis in white matter tracts. Iba1 expression was significantly increased in the optic tract of injured mice compared with sham (**A** and **B**). Red arrowhead indicates microglia. Brain coronal view: 1 mm; Scale bar for inset (enlarged view of the optic tract) = 100 μm. Analysis of A and B by unpaired t-test **(C)**; Sham (*n* = 15), rmTBI (*n* = 20). Glial fibrillary acidic protein (GFAP) expression analysis (**D** and **E**; **G** and **H**). Blue arrowhead indicates astrocytes. Brain coronal view: 1 mm; Scale bar for inset (enlarged view of the optic tract and corpus callosum) = 100 μm. Unpaired t-test analyses of D and E **(F)** and G and H **(I)**, mean ± standard deviation; Sham (*n* = 15), rmTBI (*n* = 19).

Identification of areas showing profound functional connectivity deficits in the absence of broad morphological damage prompted additional examination. Thus, we measured changes in protein levels in these areas of interest. As expected, physical damage to the optic tract coincided with an inflammatory response reflected by robust Iba1 and GFAP signals (Fig. 4A, 4B and 4D, 4E). Interestingly, we also detected strong GFAP signal in the corpus callosum (Fig. 4G, 4H), a region that did not present microstructural damage in the DTI analysis. Iba1-positive microglia were significantly increased (*p* < 0.0001) in the optic tract of injured mice (Fig. 4C). Similarly, GFAP-positive signal was significantly increased in both the optic tract (*p* < 0.0001; Fig. 4F) and corpus callosum (*p* = 0.0320; Fig. 4I) of the rmTBI.

**FIG. 5.**
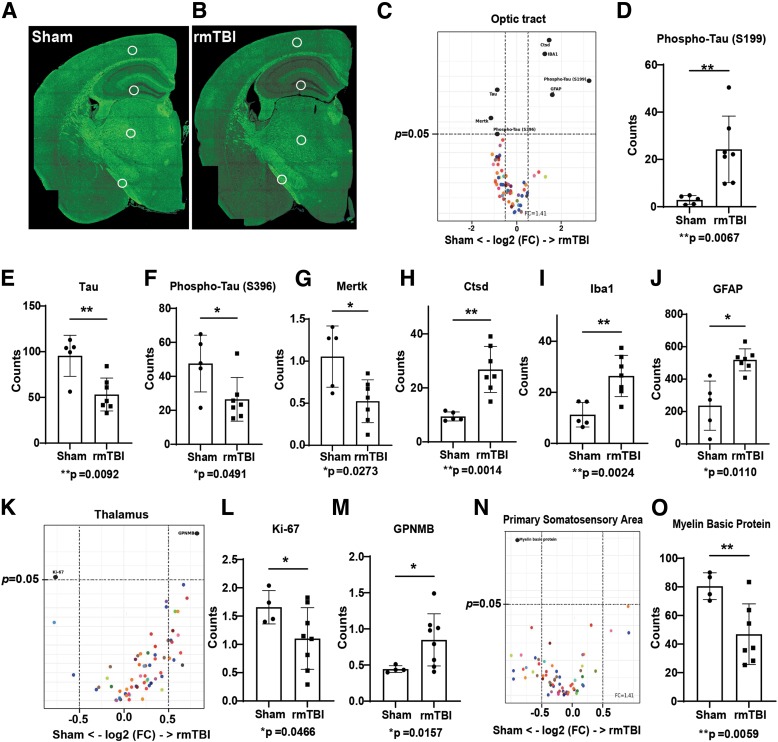
Repetitive mild traumatic brain injury (rmTBI) increased the expression of disease associated proteins. Selected regions of interest (primary somatic sensory area, dentate gyrus polymorph layer, thalamus, and the optic tract) for NanoString GeoMx spatial protein profiling analysis displayed on coronal sections of sham and rmTBI experimental animal tissue (**A** and **B,** respectively). Volcano plots **(C)** and bar graphs displaying changes in the quantity of Alzheimer's disease (AD) pathological **(D-F)** and cell markers **(G-J)** in the optic tract. Volcano plot **(K)** and bar graphs (**L** and **M**) displaying changes in the quantity of AD pathological and cell markers in the thalamus. Volcano plot **(N)** and analysis of changes to myelin basic protein levels in experimental animals following sham or rmTBI **(O)**. Protein levels were normalized to the expression of housekeeping proteins. Unpaired t-test with Welch's correction, mean ± standard deviation, *n* = 5-8.

For more extensive investigation of proteins, we used the NanoString GeoMx DSP platform. We determined that ADRD-associated proteins were significantly changed in three areas of interest identified by our rsfMRI and DTI analyses: the primary somatosensory area, thalamus, and optic tract (Fig. 5A, 5B; Table 1). We observed no changes in the dentate gyrus polymorph layer (negative data not shown). Interestingly, both total tau and phospho-tau S396 (pS396) levels were significantly decreased, but levels of one phospho-tau species, pS199 tau, were increased in the rmTBI group (Fig. 5C-F). Various neuroinflammatory markers such as Iba1, GFAP, Cathepsin-D (Fig. 5H-J) were significantly increased in the optic tract of the rmTBI group. The expression of neuroinflammatory marker glycoprotein non-metastatic melanoma protein B (GPNMB) was also significantly increased, and cell proliferation marker Ki-67 was significantly decreased in the thalamic region (Fig. 5K-M). Myelin basic protein (MBP) was significantly decreased in the primary somatosensory area, which reflects white matter damage (Fig. 5N, 5O).

**Table. 1:** Summary of the Expression of Various Neuropathological Markers Following rmTBI Identified Through NanoString GeoMx Spatial Protein Profiling Analysis

ROIs	Protein biomarker	Expression	Full target name	Cell profiling panel	*p* value
Primary somatosensory area	Myelin basic protein	Down	Myelin basic protein	Neural cell Profiling core	0.0059
Thalamus	GPNMB	Up	Glycoprotein non-metastatic melanoma protein B	Glial cell subtyping module	0.0157
	Ki-67	Down	Marker of proliferation Ki-67	Neural cell profiling core	0.0466
Optic tract	CTSD	Up	Cathepsin D	Glial cell subtyping module	0.0014
	Iba1	Up	Allograft inflammatory factor 1	Neural cell profiling core	0.0024
	pTau (S199)	Up	Microtubule-associated protein tau	AD pathology extended	0.0067
	GFAP	Up	Glial fibrillary acidic protein	Neural cell profiling core	0.0110
	Tau	Down	Microtubule-associated protein tau	AD pathology module	0.0092
	MERTK	Down	MER proto-oncogene, tyrosine kinase	Glial cell subtyping module	0.0273
	pTau(S396)	Down	Microtubule-associated protein tau	AD pathology extended	0.0491

rmTBI, repetitive mild traumatic brain injury.

## Discussion

The long-term goal of our project is to gather mechanistic information to connect head injuries to neurodegenerative disorders. Here, we identified that rmTBI altered brain functional connectivity, induced imaging abnormalities, and modified the levels of disease-associated proteins listed in Table 1. The observed changes were consistent across individually affected brain regions and may thus represent a common physiological response to rmTBI. Indeed, some markers are more transient, and more exhaustive longitudinal studies could reveal how these protein networks contribute to chronic neurodegenerative disorders. Investigating brain networks using rsfMRI and graph theory offer functional measures, such as the ones described herein, that have potential for improving diagnostic efforts for TBI and other disorders.^33-35^ Our work improves upon previous studies by dissecting more granular aspects of functional connectivity in a mouse model of rmTBI and at ultra-high field imaging.^29,36-38^

Functional metrics such as clustering coefficient, path length, centrality, and efficiency are quantifiable features that reveal how information flows throughout the network^39^; these signals were impaired following rmTBI (Fig. 2 and Fig. 3). Our findings are concordant with other pre-clinical models of mild head injury that found local alterations in clustering coefficient and local efficiency; moreover, global changes were intact,^40^ showing consistency across milder and repetitive TBI models.^41,42^ Although we did not detect global changes in clustering coefficient (also reported recently by Boroda and colleagues),^43^ the local clustering coefficient at 10% edge density was significantly reduced in the visual anteromedial area and various thalamic nuclei after rmTBI. We also observed a significant decrease in net global and local efficiency in the rmTBI group at thresholds below 10% densities; this is consistent with previous studies.^41,42^ The results reveal that local integration, efficiency, and integrative processing across distal brain regions are significantly altered following rmTBI. As previously reported, we did not observe changes in the small-world index and net clustering coefficient after rmTBI.^29^

Given that eigenvector centrality scores in the contralateral cortex nodes can be restored from 2 to 30 days after injury,^29^ it will be important to assess these metrics at later post-injury periods. Confirming these changes at different timepoints will provide valuable information about compensatory mechanisms and reorganization of damaged networks after TBI.

Consistent with previous reports,^35^ our results show that rmTBI damages thalamic nuclei. Given its numerous roles in integrating brain signaling, damage to the thalamus may result in varied manifestations after TBI; this may underlie broad symptomatology of individuals who sustained one or more TBIs. In addition to the network-associated changes, aberrant signaling at the molecular level is a probable pathogenic factor in the transition from rmTBI to chronic neurodegeneration. We observed significant changes in distinct proteins that aligned with unique brain regions. For example, accumulation of Iba1 and GFAP in the optic tract indicates that rmTBI induces gliosis; these results are consistent with our reported IHC findings as well as previous CHIMERA studies.^11^ In addition, GPNMB was significantly increased in the thalamus of the rmTBI group. GPNMB is highly expressed in glia, and is linked with risk for Parkinson's disease.^44,45^

Given their limited and unclear molecular links with TBI, follow-up experiments evaluating the prognostic value of GPNMB, Ki67, and cathepsin-D after rmTBI could unveil new biomarkers. Interestingly, total and pS396 tau levels were decreased in the optic tract, while pS199 tau was increased. This could be the result of tau cleavage resulting from head injury; importantly, it suggests that distinct post-translationally modified tau species could extravasate and reflect the state of injuries thereby serving as reliable biomarkers.^7^

The outcomes of the present study provide important information about the effect of rmTBI and its primary molecular mechanism on the underlying pathology at 5-7 dpi; this period corresponds to the secondary injury phase, where complex molecular pathways obscure predictive outcomes. Importantly, this work is limited to a single time-point, which inherently will fail to capture transient effects of rmTBI. Another limitation is the small sample size for female mice, which did not permit evaluation of sex as a biological variable. Although previous studies do not find rsfMRI differences between sexes, studies to overcome these limitations after head injury are currently underway.

Changes in the 5-7 dpi time-point could precede the long-term consequences of rmTBI. Our results could connect injuries with the pathophysiology of chronic TBI-associated neurodegenerative diseases. Our future work on the assessment of post-TBI effects, together with behavioral outcomes, will help to better understand the molecular mechanisms of TBI and their long-term negative consequences on cognition.

## Conclusion

Using highly translational imaging modalities and digital spatial protein profiling we report abnormalities following CHIMERA-induced rmTBI at 5-7 dpi. These measures may serve as potential biomarkers that can diagnose rmTBI and predict clinical outcomes. Moreover, protein changes could be responsible for the molecular pathways involved in the acute and chronic consequences of rmTBI. Studies to identify the longevity of these abnormalities and their association with cognitive dysfunction are currently underway.

## Data Access Statement

We are committed to sharing the products of our research as widely and freely as possible. Thus, we will adhere to the NIH Grant Policy on Sharing of Unique Research Resources, including the Principles and Guidelines for Recipients of NIH Research Grants and Contracts on Obtaining and Disseminating Biomedical Research Resources (December 23, 1999). Data and code are uploaded to local University of Florida network computers and HiPerGator supercomputer. Data and code will be made immediately available after publication, in accordance with University policies. They will be accessible via permission to transfer by the corresponding author. Globus or similar large file transfer programs can be used to achieve seamless transfer to requesting investigators. If any intellectual property arises which requires a patent, we will ensure that the technology (materials and data) remains widely available to the research community in accordance with the NIH Principles and Guidelines document.

## Transparency, Rigor, and Reproducibility Summary

For imaging studies, the total sample size was 18 (*n* = 8 sham; *n* = 10 rmTBI). For IHC experiments, the total sample size was 34 (*n* = 15 sham; *n* = 19 rmTBI; two batches combining injured and sham in each batch, ∼12 months apart); for NanoString experiments, the total sample size was 12 (*n* = 5 Sham, *n* = 8 rmTBI). Studies were designed to be blinded, such that the individuals providing the mice, those performing the injuries and imaging, and the data analyst were blinded to the identities of the mice; however, imaging modules revealed inflammation, allowing the possibility that the experimenter and data analyst distinguish subjects from both groups. Data were submitted in a BioRxiv preprint.

## Acknowledgments

The preprint version of this manuscript has been published and available in bioRxiv website of the preprint server: https://www.biorxiv.org/content/10.1101/2022.09.21.508917v1; doi: https://doi.org/10.1101/2022.09.21.508917

## Supplementary Material

Supplemental data

Supplemental data

Supplemental data
