# Supplementary material for: Fixed Time-Point Analysis Reveals Repetitive Mild Traumatic Brain Injury Effects on Resting State Functional Magnetic Resonance Imaging Connectivity and Neuro-Spatial Protein Profiles
Source: J Neurotrauma. 2023 Sep 29;40(19-20):2037–49. doi: 10.1089/neu.2022.0464 (PMC10541943; doi:10.1089/neu.2022.0464)

**Fig. S1.** Representative DTI brain image shows ROIs selected for microstructure pattern analysis.


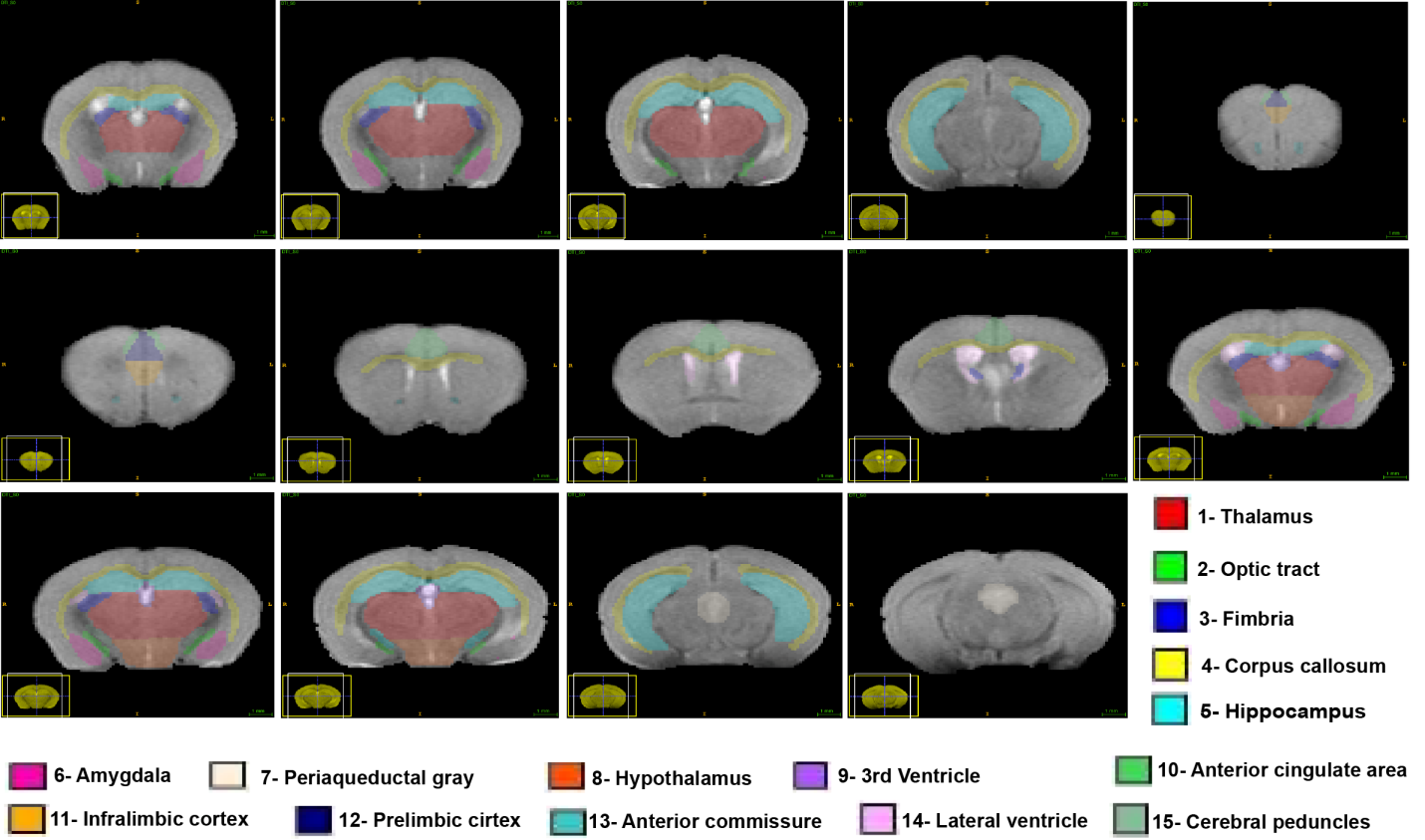

Supplement: Supplemental data [file Suppl_FigS1.docx]
