# Supplementary material for: Fixed Time-Point Analysis Reveals Repetitive Mild Traumatic Brain Injury Effects on Resting State Functional Magnetic Resonance Imaging Connectivity and Neuro-Spatial Protein Profiles
Source: J Neurotrauma. 2023 Sep 29;40(19-20):2037–49. doi: 10.1089/neu.2022.0464 (PMC10541943; doi:10.1089/neu.2022.0464)
Supplement: Supplemental data [file Suppl_TableS1.docx]

**Supplementary table. 1.** The complete list of 64 ROIs (left and right) analyzed through rsfMRI and graph theory.

| **ROI #** | **Abbreviation** | **Region** |
| --- | --- | --- |
| 1 | L VISAA | Visual Anterior area |
| 2 | L VISRLA | Visual Rostrolateral area |
| 3 | L VISAMA | Visual Anteromedial area |
| 4 | L VISLA | Visual Lateral area |
| 5 | L VISPV1 | Visual primary visual |
| 6 | L VISPV2 | Visual primary visual |
| 7 | L VISPL | Visual Posterolateral |
| 8 | L VISPM | Visual Posteromedial |
| 9 | L VISPR | Visual postrhinal |
| 10 | L THA MED | Thalamus mediodorsal |
| 11 | L THA L | Thalamus anterolateral complex of ventral thalamus |
| 12 | L THA AC | Thalamus laterodorsal |
| 13 | L THA PC | Thalamus posterior complex |
| 14 | L THA VP | Thalamus ventral posteromedial |
| 15 | L THA para | Thalamus paracentral nucleus |
| 16 | L THA PL | Thalamus posterior lateral |
| 17 | L THA CL | Thalamus central lateral – parafascicular |
| 18 | L THA AP | Thalamus anterior pretectal nucleus |
| 19 | L THA DL | Thalamus dorsal lateral geniculate |
| 20 | L THA V | Thalamus ventromedial |
| 21 | L TEMP | Temporal association areas |
| 22 | L DEN | dentate nuclei of cerebellum |
| 23 | L CERE | interposed complex of cerebellum |
| 24 | L CP G | genu of corpus callosum |
| 25 | L CP1 | Body of corpus callosum 1 |
| 26 | L CP2 | Body of corpus callosum 2 |
| 27 | L SCP1 | splenium of corpus callosum 1 |
| 28 | L SCP2 | splenium of corpus callosum 2 |
| 29 | L SCP3 | splenium of corpus callosum 3 |
| 30 | L HYPTH | Hypothalamus |
| 31 | L OT | Optic tract |
| 32 | L CERP | Cerebral peduncles |
| 33 | R VISAA | Visual Anterior area |
| 34 | R VISRLA | Visual Rostrolateral area |
| 35 | R VISAMA | Visual Anteromedial area |
| 36 | R VISLA | Visual Lateral area |
| 37 | R VISPV1 | Visual primary visual |
| 38 | R VISPV2 | Visual primary visual |
| 39 | R VISPL | Visual Posterolateral |
| 40 | R VISPM | Visual Posteromedial |
| 41 | R VISPR | Visual postrhinal |
| 42 | R THA MED | Thalamus mediodorsal |
| 43 | R THA L | Thalamus anterolateral complex of ventral thalamus |
| 44 | R THA AC | Thalamus laterodorsal |
| 45 | R THA PC | Thalamus posterior complex |
| 46 | R THA VP | Thalamus ventral posteromedial |
| 47 | R THA para | Thalamus paracentral nucleus |
| 48 | R THA PL | Thalamus posterior lateral |
| 49 | R THA CL | Thalamus central lateral – parafascicular |
| 50 | R THA AP | Thalamus anterior pretectal nucleus |
| 51 | R THA DL | Thalamus dorsal lateral geniculate |
| 52 | R THA V | Thalamus ventromedial |
| 53 | R TEMP | Temporal association areas |
| 54 | R DEN | dentate nuceli of cerebellum |
| 55 | R CERE | interposed complex of cerebellum |
| 56 | R CP G | genu of corpus callosum |
| 57 | R CP1 | Body of corpus callosum 1 |
| 58 | R CP2 | Body of corpus callosum 2 |
| 59 | R SCP1 | splenium of corpus callosum 1 |
| 60 | R SCP2 | splenium of corpus callosum 2 |
| 61 | R SCP3 | splenium of corpus callosum 3 |
| 62 | R HYPTH | Hypothalamus |
| 63 | R OT | Optic tract |
| 64 | R CERP | Cerebral peduncles |
