# Supplementary material for: Fixed Time-Point Analysis Reveals Repetitive Mild Traumatic Brain Injury Effects on Resting State Functional Magnetic Resonance Imaging Connectivity and Neuro-Spatial Protein Profiles
Source: J Neurotrauma. 2023 Sep 29;40(19-20):2037–49. doi: 10.1089/neu.2022.0464 (PMC10541943; doi:10.1089/neu.2022.0464)

**Fig. S2. Network disruption by rmTBI is not widespread.** No differences in the Network strength **(A)**, Louvain modularity **(B)**, Assortativity **(C)**, Transitivity **(D)**, and Gamma **(E)**. Net lambda **(F)** was significantly increased in rmTBI group at 4%, 6% and 10% density threshold levels compared to sham. The global network metrices were analyzed by 2-way ANOVA, mean ± standard error at edge densities ranges 2 to 40%.


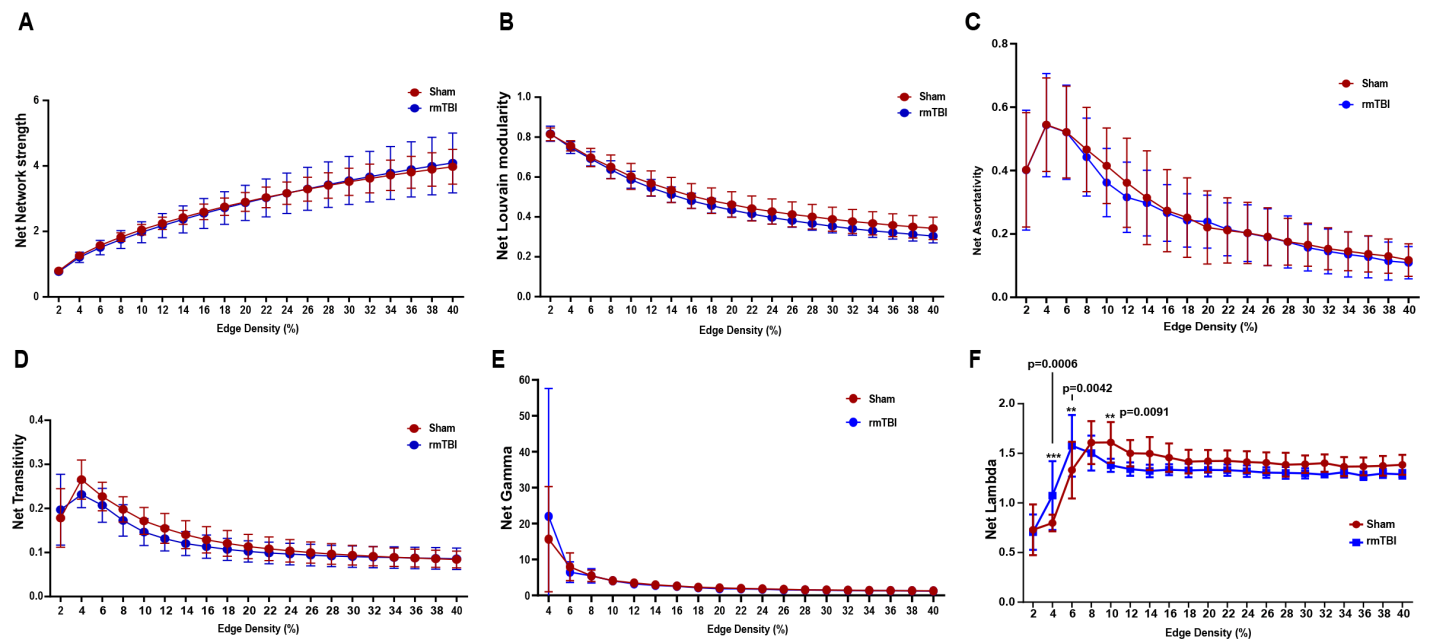

Supplement: Supplemental data [file Suppl_FigS2.docx]
